# The influence of HIV infection on myocardial fibrosis diagnosed by cardiac magnetic resonance imaging in adults: a systematic review and meta-analysis of observation studies

**DOI:** 10.3389/fcvm.2025.1534533

**Published:** 2025-01-29

**Authors:** Katongo Hope Mutengo, Bruno Bezerra Lima, Wilbroad Mutale, Aggrey Mweemba, Lorrita Kabwe, Clive Banda, Callistus Kaayunga, Mutale Mulenga, Douglas Heimburger, Sepiso K. Masenga, John Jeffrey Carr, Annet Kirabo

**Affiliations:** ^1^ Department of Internal Medicine, Ministry of Health, Monze Mission Hospital, Monze, Zambia; ^2^ Mulungushi University School of Medicine and Health Sciences, HAND Research Group, Livingstone, Zambia; ^3^ Division of Cardiology, Vanderbilt University Medical Center, Nashville, TN, United States; ^4^ School of Public Health, University of Zambia, Lusaka, Zambia; ^5^ Ministry of Health, Nephrology Unit, University Teaching Hospital Lusaka Adult Hospital, Lusaka, Zambia; ^6^ Department of Adult Cardiology, Ministry of Health, National Heart Hospital, Lusaka, Zambia; ^7^ Ministry of Health, Southern Province Health Office, Choma, Zambia; ^8^ Vanderbilt University Medical Center, Vanderbilt Institute for Global Health, Nashville, TN, United States; ^9^ Department of Radiology and Radiological Sciences, Vanderbilt University Medical Center, Nashville, TN, United States; ^10^ Department of Molecular Physiology and Biophysics, Vanderbilt University Medical Center, Nashville, TN, United States

**Keywords:** myocardial, fibrosis, HIV, CMR, LGE, ECVF, T1 mapping

## Abstract

**Introduction:**

Human immunodeficiency virus (HIV) infection is linked to myocardial fibrosis. Observational studies using cardiac magnetic resonance (CMR) have explored this relationship but scarcity of data synthesis limits our understanding. Our systematic review and meta-analysis aimed to synthesize associations between HIV and myocardial fibrosis from CMR-based observational studies in adults.

**Methods:**

We identified 12 studies (2013–2024) with 1,769 participants [1,117 people with HIV (PWH)]. Three studies were cohort and nine were cross-sectional. Meta-analysis included seven studies on late gadolinium enhancement (LGE) (1,081 participants: 669 PWH), eight on native T1 mapping (840 participants: 467 PWH), and ten on ECVF (1,603 participants: 992 PWH). We examined myocardial fibrosis prevalence via the prevalence difference in LGE, and severity by mean differences in native T1 mapping values [milliseconds (ms)] and global extracellular volume fraction (ECVF,%) between PWH and HIV-uninfected individuals, using random effects model.

**Results and discussion:**

Pooled analyses showed PWH had a 33% higher prevalence of LGE (95% CI: 12.0%–54.0%, I^2^ = 94.5%, *p* < 0.001), a mean native T1 mapping difference of 27.30 ms (95% CI: 11.21–43.39 ms, I^2^ = 88.2%, *p* < 0.001), and a mean ECVF difference of 1.85% (95% CI: 0.63%–3.08%, I^2^ = 90.5%, *p* < 0.001), respectively. Meta-regression showed no significant associations between ECVF and demographic, HIV-related, or cardiac factors. LGE and native T1 mapping analyses lacked sufficient data for meta-regression. In conclusion, PWH exhibit significantly higher prevalence and severity of myocardial fibrosis compared to HIV-uninfected individuals. But standardized methodologies and further research are essential to enhance consistency.

**Systematic Review Registration:**

https://www.crd.york.ac.uk/prospero/display_record.php?RecordID=533379, CRD [42024533379].

## 1 Introduction

Human immunodeficiency virus (HIV) is a global health challenge affecting millions worldwide. Beyond its well-known immunological impact, there's growing concern regarding cardiovascular complications associated with HIV, particularly myocardial fibrosis (1–3). Myocardial fibrosis, characterized by an overabundance of extracellular matrix proteins (ECM) within the myocardium (4–8), occurs more frequently in people with HIV (PWH) (1–3, 9, 10). Myocardial fibrosis manifests as interstitial fibrosis or replacement fibrosis, with the former associated with diffuse ECM deposition (2) and the latter replacing dead cardiomyocytes following injury, typically a myocardial infarction (5, 11, 12). Chronic inflammation, immune activation, and potential anti-retroviral treatment (ART) cardiotoxicity are implicated in HIV-related myocardial fibrosis (2). While myocardial fibrosis is often silent in its initial stages, it carries significant clinical implications, as it can predispose affected individuals to potentially life-threatening cardiac arrhythmias and sudden cardiac death (6–8). Therefore, early detection of subclinical myocardial fibrosis is crucial for navigating adverse cardiovascular outcomes in PWH but the invasive nature of gold-standard histology limits its clinical utility.

Cardiac magnetic resonance (CMR) offers a non-invasive, accurate alternative for diagnosing myocardial fibrosis (9). CMR is a versatile, non-invasive tool that measures critical aspects of myocardial fibrosis and inflammation (11). Despite its potential, current literature presents varied conclusions on the relationship between HIV and CMR-diagnosed myocardial fibrosis. For instance, a study by Shuldiner et al. (10) in South Africa found greater CMR-diagnosed myocardial fibrosis in asymptomatic PWH, particularly among women, suggesting a significant impact of HIV on myocardial fibrosis in female patients. Conversely, a U.S. study by Williams et al. (13) on adults with perinatally acquired HIV found no significant association between HIV and diffuse myocardial fibrosis, highlighting the possible influence of HIV acquisition mode and ART timing on cardiovascular outcomes. These contrasting findings underscore the complexity of myocardial fibrosis in HIV patients, reflecting the diversity of study populations and methodologies.

Various imaging techniques are employed with CMR to assess the different types of myocardial fibrosis. Among them, the late gadolinium enhancement (LGE) technique is particularly effective in detecting replacement fibrosis, which is indicative of areas where dead cardiomyocytes have been replaced with fibrotic tissue (14). It also correlates strongly with histological findings (15). On the other hand, T1 mapping is effective for identifying interstitial fibrosis, which involves diffuse ECM deposition (12). The extracellular volume fraction (ECVF) measurement complements these techniques by assessing the volume of extracellular space, providing a comprehensive view of diffuse myocardial fibrosis (16). Combining these modalities enhances the ability to differentiate between various types of myocardial fibrosis and improves diagnostic accuracy. Our review aims to synthesize data from observational studies to provide a comprehensive understanding of the relationship between HIV and the prevalence and severity of myocardial fibrosis in adults as diagnosed by CMR, utilizing LGE, native T1 mapping and ECVF.

## 2 Materials and methods

### 2.1 Literature search

Our review involved searching databases including PubMed, Embase, Scopus, and Web of Science, supplemented by manual searches in the reference lists of included articles and relevant reviews, updated until the point of manuscript submission. We used keywords and phrases associated with “HIV”, “myocardial fibrosis”, “cardiac fibrosis”, and “cardiac magnetic resonance imaging” (CMR or Cardiac MRI), including “extracellular volume fraction” (ECVF), and “T1 mapping”. Additional terms included variations related to “HIV”, such as “PWH” (People with HIV), “PLWHIV” (People Living with HIV), “PLHIV” (People Living with HIV), and “AIDS' (Acquired Immunodeficiency Syndrome). For CMR parameters, synonyms like “Delayed Enhancement” for LGE, “Extracellular Fraction” (ECF) or “Extracellular Volume (ECV) for ECVF”, and “Longitudinal Relaxation Time Mapping” for T1 mapping were also incorporated. Boolean operators such as AND, OR, NOT were employed to refine the search, incorporating relevant synonyms and abbreviations. Our search was limited to English-language publications, with no restrictions on region, publication date or status.

### 2.2 Eligibility criteria

Articles were included if the study design was observational and the population consisted of adults (≥18 years) with PWH or HIV-uninfected individuals. The diagnosis of myocardial fibrosis had to be conducted using CMR and report quantitative measures of myocardial fibrosis, including the frequency of participants with LGE and global ECVF quantification expressed as percentages, as well as native T1 mapping in milliseconds (ms). We considered all studies examining ECVF utilizing the Modified Look-Locker Inversion recovery (MOLLI) or just Look-Locker Inversion recovery technique used measure diffuse myocardial fibrosis by quantifying ECVF (using hematocrit measurements), as well as LGE for assessing myocardial scarring or replacament fibrosis. We also considered all studies employing CMR scanners with field strengths ranging from 1.5 Tesla (T) to 3 T to assess parameters including myocardial fibrosis, myocardial inflammation, ventricular function, and tissue characterization. Details of the CMR machines utilized including the CMR protocol utilized for the studies can be found in Supplementary file 1 Table 1. Studies were excluded if they focused on other diagnostic methods such as CT-imaging, echocardiography, histology, or serum biomarkers, or did not provide sufficient data to compute effect sizes. Additionally, review articles, letters, editorials, case reports, and conference abstracts were excluded. When multiple reports stemmed from the same study, we selected only the most recent or comprehensive one for our meta-analysis. For instance, between the publications by Holloway et al. (9) and Ntusi et al. (1), we included the latter in our analysis.

### 2.3 Data extraction

We extracted relevant information from the included studies using a pre-designed data extraction form and the electronic data capture tool REDCap, later saved in an Excel spreadsheet. The extracted data included study characteristics such as authors, publication year, study location, and study design; participant demographics and clinical parameters [blood pressure, body mass index (BMI), lipid profiles, glucose levels, etc.]; cardiac functional and structural changes [left ventricular end diastolic volume (LVEDV, ml), left ventricular ejection fraction (LVEF,%), left ventricular mass index (LVMi)]; and HIV-related factors like ART exposure and duration of HIV infection. Details of CMR assessment, specifically measurements of LGE, ECVF, and native T1 mapping, were also recorded. ECVF reported as a fraction in some studies was converted to a percentage maintaining the same values. All units for lipid profiles or blood glucose were converted into SI units (mmol/L) if they were reported in mg/dl using a validated MDApp conversion calculator (link: https://www.mdapp.co/cholesterol-conversion-calculator-600/). For cohort studies, we used the final outcome as our data collection point to allow the consistent in data with the cross-sectional studies.

### 2.4 Quality assessment

Two reviewers (KHM and MM) independently performed the quality assessment using the Newcastle-Ottawa Scale (NOS) for cohort and case-control studies. Each study was evaluated on three broad perspectives: the selection process of the study groups (0–4 points), the comparability of the groups (0–2 points), and the ascertainment of either the exposure or outcome of interest (0–3 points). Studies were first evaluated according to established questions, scored as 1 if the item was considered in the study or 0 if it was not considered or if it was impossible to determine whether it was considered. We assigned scores of 0–3, 4–6, and 7–9 points for low, moderate, and high-quality studies, respectively (Supplementary file 1 Table 2). Any discrepancies in quality assessment were resolved through discussion. These discussions focused on the specific criteria where differences occurred, supported by referencing the quality assessment tool and relevant study details. In cases where agreement could not be reached through discussion, the third reviewer (SM) independently reviewed the conflicting assessments and provided their input. The group then collectively considered the third reviewer's judgment, which often helped highlight overlooked aspects or clarified ambiguities.

### 2.5 Statistical analysis

We analyzed the data qualitatively and quantitatively to summarize our findings. The quantitative data was analyzed using Meta package in Stata (StataCorp. 2021. Stata Statistical Software: Release 17. College Station, TX: StataCorp LLC).

#### 2.5.1 Meta-analysis for prevalence of myocardial fibrosis (LGE)

We aggregated the results of the prevalence myocardial fibrosis from the total number of participants with LGE out of the total number who underwent LGE-CMR reported in the included studies. The effect size for each study was determined as the prevalence difference of LGE among the PWH and HIV-uninfected groups using percentages. Primary outcome data encompassed the presence of LGE. The prevalence differences were synthesized using a random-effects inverse-variance model with the DerSimonian-Laird (DL) of τ^2^ (tau squared) to account for observed variability between studies. The DL estimator was chosen for the primary analysis due to its computational efficiency and widespread use in similar meta-analyses. To address potential concerns regarding the robustness of our findings and the estimation of variance components, we conducted sensitivity analyses using Residual Maximum Likelihood (REML) in meta-regression models. We assessed heterogeneity using I^2^ statistic, and τ^2^, with I^2^ values indicating the degree of variability attributable to heterogeneity rather than chance. We used an I^2^ < 50% to indicate low heterogeneity and I^2^ of 50% and above to indicate high heterogeneity.

#### 2.5.2 Meta-analysis for severity of myocardial fibrosis (native T1 mapping and ECVF)

We calculated the mean differences in native T1 mapping values and ECVF between PWH and HIV-uninfected participants. The primary outcome variables were the mean difference of ECVF and native T1 mapping values between PWH and HIV-uninfected participants. These mean differences were synthesized using a random-effects inverse-variance model with the DL estimator of τ^2^. This approach allowed us to assess the average difference in the severity of myocardial fibrosis between the two groups. Heterogeneity was assessed using similar statistical measures as for the prevalence data.

#### 2.5.3 Meta-regression analysis

We examined several demographic and clinical covariates to assess their association with myocardial fibrosis outcomes (LGE, native T1 mapping, and ECVF) among PWH and HIV-uninfected individuals. However, we observed significant variability in the covariates reported across studies for LGE and native T1 mapping. This variability resulted in insufficient pooled data, leading us to exclude these outcomes from the meta-regression analyses. Consequently, we focused our meta-regression analysis on ECVF, selecting covariates that were consistently reported across studies. Furthermore, we performed a leave-one-out sensitivity analysis using REML to assess the robustness of our findings by re-running the meta-regression to evaluate the impact of each individual study on the overall results. The three models we employed included: Model 1: Age, sex distribution (number of males and females), left ventricular end-diastolic volume (LVEDV), and left ventricular ejection fraction (LVEF). Model 2: Lipid profiles - total cholesterol, high-density lipoprotein (HDL) cholesterol, and low-density lipoprotein (LDL) cholesterol. Model 3: Duration of antiretroviral therapy (ART) and CD4 count. These combinations were chosen because while they were our covariates of interest, they also provided sufficient data for analysis. Other covariates of interest, such as body mass index (BMI), blood pressure measurements, left ventricular mass index (LVMi), hematocrit count, triglycerides, blood glucose levels, smoking status, hypertension, diabetes mellitus, ART regimen, and duration of HIV infection, were not included in the meta-regression analyses due to a lack of sufficient data pooled data. We did not contact the authors to request additional data. This decision was based on the observation that many of these studies explicitly acknowledged small sample sizes as a limitation (1, 10, 13, 17–21). Given these constraints, it was unlikely that additional data would sufficiently address the issues of insufficient information for computing effect sizes. For all analyses, weighted standard errors were used to account for variability in the precision of the estimates across studies, allowing for a more accurate assessment of the relationship between the covariates and the outcome. A *p*-value less than 0.05 was considered statistically significant for all analyses.

## 3 Results

### 3.1 Study selection

**Figure 1:**
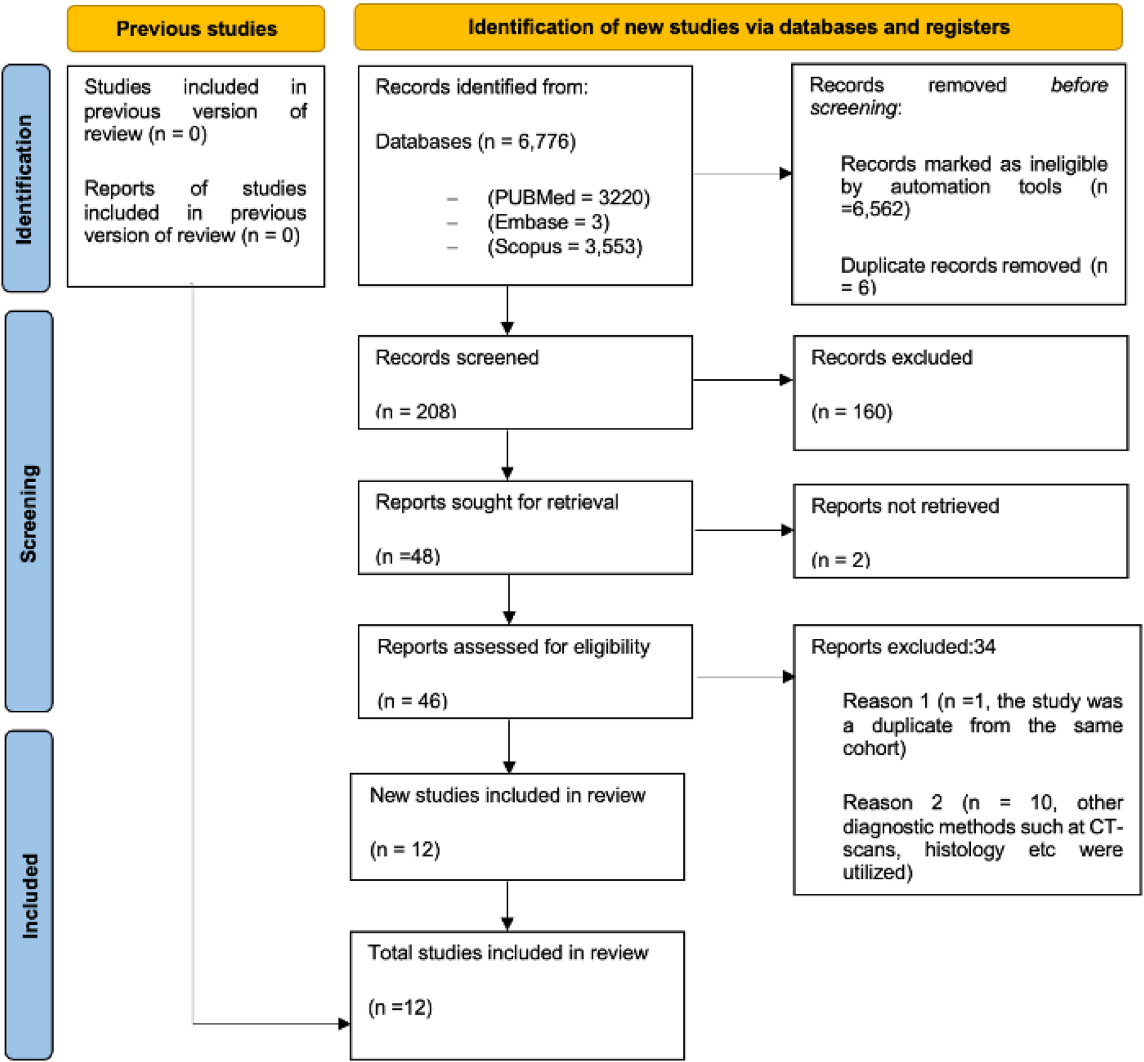
The preferred reporting items for systematic reviews and meta-analysis (PRISMA) flow chart showing the systematic approach for the selection process of studies for inclusion. PRISMA, preferred reporting items for systematic reviews and meta-analysis.

We conducted a comprehensive literature search across major databases: PubMed (3,220 records), Embase (3 records), and Scopus (3,553 records), yielding a total of 6,776 studies. We did not successfully retrieve any studies from Web of Science. After removing 6,562 records identified as ineligible by automation tools and 6 duplicate records, 208 studies remained for initial screening. Of these, 160 records were excluded based on titles and abstracts - 59 by automation tools and 101 by human review - leaving 48 studies for which full-text retrieval was sought. Two of these could not be retrieved, leaving 46 studies to be assessed for eligibility. Upon detailed evaluation, 34 studies were excluded for the following reasons: (1) 1 study was excluded as it was a duplicate from the same cohort, but with different time points; the latest publication with additional participants was selected, (2) 10 studies used diagnostic methods other than CMR, such as CT scans or histology, and (3) 23 studies were excluded due to inadequate data on outcomes of interest or restricted access. Finally, 12 studies were included in the systematic review and meta-analysis (Figure 1). Of these, three (25.0%) were cohort studies, and the remaining nine (75.0%) were cross-sectional.

The studies in the dataset span from 2013 to 2024, involving a total of 1,769 participants, 1,117 (63.1%) were PWH (Table 1). The geographical distribution of the studies is as follows: six studies (50.0%) were conducted in the United States of America (USA) (13, 19, 21–24), two studies (16.7%) in South Africa (10, 18), one study (8.3%) in the United Kingdom (UK) (1), one study (8.3%) in Germany (17), one study (8.3%) in China (20), and one study (8.3%) in Peru (25).

**Table 1:** Characteristics of and findings from the included studies.

No	Type of study	Year of publication	Study location	First Author	Type of study	Total sample size (HIV infected, HIV uninfected)	Summary of findings
1	Immune correlates of diffuse myocardial fibrosis and diastolic dysfunction among aging women with human immunodeficiency virus	2019	USA	Zanni et al.	Cross-sectional	34 (20, 14)	Asymptomatic, antiretroviral-treated women with HIV exhibited increased myocardial fibrosis (ECV = 0.34 ± 0.06 vs. 0.29 ± 0.04, *P* = .002), reduced diastolic function (diastolic strain rate = 1.10 ± 0.23 s^−1^ vs. 1.39 ± 0.27 s^−1^, *P* = .003), and heightened systemic monocyte activation compared to HIV-uninfected women. Specific immune markers such as soluble CD163 and circulating inflammatory CD14+CD16+ monocyte CCR2 expression correlated with the degree of myocardial fibrosis and diastolic dysfunction.
2	Evolution of myocardial oedema and fibrosis in HIV infected persons after the initiation of antiretroviral therapy: a prospective cardiovascular magnetic resonance study	2022	South Africa	Robbertse et al.^a^	Cohort (prospective)	95 (73, 22)	Newly diagnosed, ART-naive PWH exhibited higher global native T1 (1,008 ± 31 ms vs. 1,032 ± 44 ms; *p* = 0.02), global T2 (46 ± 2 ms vs. 48 ± 3 ms; *p* = 0.006), and prevalence of pericardial effusion (18% vs. 67%; *p* < 0.001) compared to HIV uninfected. After 9 months on ART, global native T1 decreased (1,032 ± 44–1,014 ± 34 ms; *p* < 0.001), ECV decreased (26% ± 4%–25% ± 3%; *p* = 0.001), and replacement fibrosis was significantly higher (49% vs. 10%; *p* = 0.02). However, the prevalence of LGE did not change significantly at 9 months compared to baseline in PWH (49% vs. 55%; *p* = 0.4).
3	Circulating biomarker correlates of left atrial size and myocardial extracellular volume fraction among persons living with and without HIV	2022	USA	Peterson et al.	Cohort (prospective)	381 (235, 146)	Myocardial ECVF was slightly elevated in PWH compared to controls (28.7% vs. 28.2%, *p* = 0.03). This indicates a small but significant increase in myocardial fibrosis in PWH relative to HIV-uninfected individuals. PWH population also had higher levels of sCD14, GDF-15, and NT-proBNP compared to HIV-uninfected but they minimally reflected HIV-associated elevations in left atrial volume index (LAVI) and myocardial ECVF
4	Myocardial fibrosis among antiretroviral therapy-treated persons with human immunodeficiency virus in South Africa	2020	South Africa	Shuldiner et al. (10)	Cross-sectional	229 (134, 95)	Compared to HIV-uninfected controls, PWH exhibited greater myocardial fibrosis, with an absolute difference in ECVF of 1.2% (95% CI: 0.1%–2.3%). Subgroup analyses showed that this effect was more pronounced in women, with women also having higher odds of elevated NT-proBNP levels (>125 pg/ml; OR: 2.4, 95% CI: 1.0–6.0). Among all PWH, elevated NT-proBNP levels were associated with significantly higher ECV (3.4% higher; 95% CI: 1.3–5.5). These findings suggest that HIV may contribute to increased myocardial fibrosis, particularly among women.
5	HIV-1-related cardiovascular disease is associated with chronic inflammation, frequent pericardial effusions, and probable myocardial edema	2016	United Kingdom	Ntusi et al. (1)	Cross-sectional	195 (103, 92)	Ntusi et al. reported 83% of PWH had LGE for myocardial fibrosis vs. 16% in controls (*p* < 0.001). HIV-infected individuals had 6% lower left ventricular ejection fraction (*P* < 0.001), 7% higher myocardial mass (*P* = 0.02), 29% lower diastolic strain rate (*P* < 0.001), and higher native T1 values (969 vs. 956 ms, *P* = 0.01). Myocardial fibrosis and pericardial effusions were 4 and 3 times more common in PWH (both *P* < 0.001).
6	Cardiac magnetic resonance reveals signs of subclinical myocardial inflammation in asymptomatic HIV-infected patients	2016	Germany	Luetkens et al. (17)	Cohort prospective	50 (28, 22)	82.1% of PWH had myocardial fibrosis vs. 27.3% in controls (*P* < 0.001). PWH showed lower LVEF (60.9% vs. 65.2%, *P* = 0.023) and lower global peak systolic strain (longitudinal strain, −17.7% vs. −20.2%, circumferential strain, −21.2% vs. −24.7%, *P* < 0.001). Elevated myocardial inflammation markers in PWH included higher native T1 (1,128.3 ms vs. 1,086.5 ms, *P* = 0.009) and early gadolinium enhancement (3.1 vs. 2.1, *P* = 0.003).
7	Human immunodeficiency viral infection and differences in interstitial ventricular fibrosis and left atrial size	2021	USA	Wu et al. (22)^b^	Cross-sectional	436 (273, 163)	PWH had a higher ECVF (29.2% vs. 28.3%, *P* = 0.04) and a larger left atrial volume (LAVI) (29.7 vs. 27.8 ml/m^2^, *P* = 0.05). ECVF was 0.84% higher in PWH after multivariate adjustment (*P* = 0.05) and LAVI was 2.45 ml/m^2^ larger (*P* = 0.01). No significant differences were found in left ventricular ejection fraction, right ventricular function, biventricular volumes, masses, LGE prevalence, or ischemic scars between PWH and controls.
8	Abnormal myocardial function is related to myocardial steatosis and diffuse myocardial fibrosis in HIV infected adults	2015	USA	Thiara et al.	Cross-sectional	125 (95, 20)	There was significantly reduced systolic function in HIV-infected subjects compared to controls (mean radial strain 21.7% vs. 30.5%, *P* = 0.004). Intramyocardial lipid levels and fibrosis index were higher in HIV-infected subjects (*P* ≤ 0.04 for both) and correlated with decreased myocardial function. Lipid levels were positively correlated with antiretroviral therapy duration and visceral adiposity. Impaired myocardial function was associated with increased levels of monocyte chemoattractant protein 1 and lipopolysaccharide binding protein.
9	Cardiac involvement in human immunodeficiency virus infected patients: an observational cardiac magnetic resonance study	2021	China	Yan et al.	Cross-sectional	68 (47, 21)	There was decreased left (55.3% vs. 63.0%, *P* < 0.001) and right (35.9% vs. 50.8%, *P* < 0.001) ventricular systolic function in PWH. Radial (30.7% vs. 39.3%, *P* = 0.001), circumferential (−17.5% vs. −19.4%, *P* = 0.008), and longitudinal (−9.4% vs. −12.8%, *P* = 0.012) systolic strains were also lower in PWH. Native T1 relaxation time (1,337.2 vs. 1,249.5 ms, *P* < 0.001), ECVF (33.5% vs. 28.5%, *P* = 0.026), and T2 relaxation time (45.2 vs. 42.0 ms, *P* = 0.001) were higher in PWH. Myocardial fibrosis was observed in 24.4% of HIV-infected patients. Advanced HIV disease was associated with lower ECVF (29.1% vs. 35.2%, *P* < 0.001) and higher frequency of LGE (8% vs. 43.8%, *P* = 0.014), and was linked to myocardial fibrosis. These findings supported the earlier initiation of ART in PWH.
10	Myocardial inflammation and edema in people living with human immunodeficiency virus	2020	Peru	Menacho et al.	Cross-sectional	72 (51, 21)	Untreated PWH had significantly higher global native T1 (1,304 vs. 1,285 ms; *P* < 0.001) and ECVF (29.3% vs. 27.7%; *P* < 0.001) compared to those on ART. Higher ECVF was associated with lower CD4 count (*r* = −0.46; *P* = 0.001) and detectable viral load, which also correlated with higher ECVF (29 vs. 27.5%; *P* < 0.001) and native T1 (1,300 vs. 1,286 ms; *P* = 0.02). The study highlights significant subclinical myocardial dysfunction and structural changes in untreated PLWH, with a notable expansion in ECVF indicating a greater myocardial impact of HIV, even in younger patients.
11	Diffuse myocardial fibrosis is uncommon in people with perinatally acquired human immunodeficiency virus infection	2024	USA	Williams et al.	Cross-sectional	40 (14, 26)	Individuals with perinatally acquired HIV did not exhibit significant diffuse myocardial fibrosis based on native T1 mapping or ECVF values compared to uninfected controls. Both groups had similar demographic data, CMR functional/volumetric data, and pre-contrast T1 mapping values. Suggesting that perinatally acquired HIV may not be associated with increased myocardial fibrosis.
12	Subclinical myocardial disease by cardiac magnetic resonance imaging and spectroscopy in healthy HIV/Hepatitis C virus-coinfected persons	2017	USA	Chew et al.^c^	Cross-sectional	18 (18, 0)	HIV-monoinfected individuals had a significantly higher median ECVF (0.30) compared to the HIV/HCV-coinfected group (0.26; *P* < 0.01), despite similar overall myocardial fat content (0.48% in both groups). HIV/HCV-coinfected participants had a greater LV mass index and LV mass/volume ratio, with median values significantly higher than those in the HIV-monoinfected group. Additionally, HIV-monoinfected individuals showed more focal myocardial scars and worse diastolic function, with 30% of HIV/HCV-coinfected patients having an abnormal mitral valve E/A ratio compared to 13% in the HIV-monoinfected group.

^a^ Out of 22, only 11 controls were reported to have LGE done in this study.

^b^ 389 participants underwent LGE-CMR.

^c^ This study was only reviewed qualitatively due to lack of HIV-uninfected controls for the outcome variables.

### 3.2 Characteristics and findings of included studies

Overall, the studies revealed a higher burden of myocardial fibrosis in PWH compared to the HIV uninfected. Ntusi et al. (1) found the highest prevalence of LGE in the UK, with 83% of PWH showing myocardial fibrosis compared to 16% in controls (*p* < 0.001), highlighting significant myocardial structural changes. On the other hand, Thiara et al. (19) found significantly reduced myocardial systolic function [mean radial strain ( ± SD), 21.7 ± 8.6% vs. 30.5 ± 14.2%; *P* = 0.004] and increased intramyocardial lipid levels (1.14% vs. 0.58%, *p* < 0.04) in PWH compared to HIV uninfected, although only 8.6% of PWH showed LGE compared to 7.7% in controls (*p* = 0.8). Yan et al. (20) in China observed the highest native T1 values (1,337.2 ms vs. 1,249.5 ms, *p* < 0.001) and ECVF (33.5% vs. 28.5%, *p* = 0.026) in PWH compared HIV uninfected, particularly in patients with AIDS. In the USA, Peterson et al. (23) reported that myocardial ECVF was slightly elevated in PWH (28.7% vs. 28.2% in controls, *p* = 0.03). Among the African population, Shuldiner et al. (10) reported greater diffuse myocardial fibrosis, with an ECVF of 30.4% compared to 29.3% in controls (*p* = 0.04). Furthermore, Zanni et al. (21) linked increased myocardial fibrosis and diastolic dysfunction in women with HIV in a USA cohort, reporting a global ECVF of 34% compared to 29% in controls (*p* = 0.002). The summary of the studies and findings are as highlighted in Table 1.

### 3.3 Meta-analysis of the prevalence of LGE in PWH and HIV-uninfected

**Figure 2:**
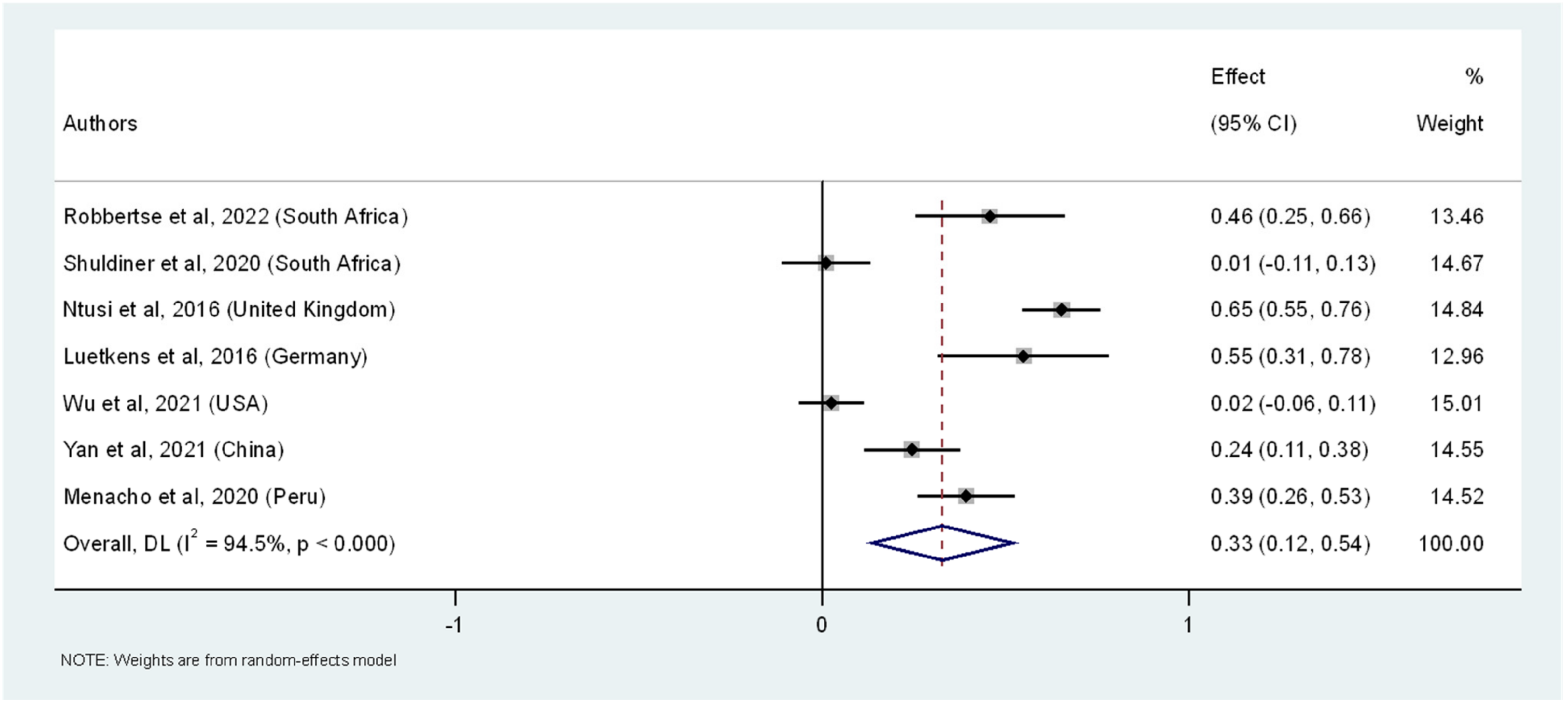
The forest plot of the prevalence difference of myocardial fibrosis, as indicated by LGE, in PWH across seven studies. The squares represent the prevalence difference estimates for each study, with horizontal lines indicating the 95% confidence intervals. The size of each square reflects the weight of the study in the meta-analysis. The diamond at the bottom represents the overall pooled prevalence and its confidence interval, derived from a random-effects model.

We included 1,081 participants (669 PWH and 412 HIV-uninfected) (Supplementary file 1 Table 3) for the prevalence difference of LGE as a marker of myocardial fibrosis. The overall pooled analysis revealed that PWH had a 33% higher prevalence of myocardial fibrosis compared to HIV-uninfected individuals (95% CI: 12.0%–54.0%, I^2^ = 94.5%, *p* < 0.001) (Figure 2). Study-specific prevalence estimates did not vary substantially, with the weights assigned to individual studies reflective of the number of PWH. Wu et al. contributed the highest weight to the overall meta-analytic effect at 15.0%, followed by Ntusi et al. with a weight of 14.8%. The least contribution was from Luetkens et al. with the overall weight contribution of 12.7%. Among the individual studies, the highest prevalence difference was observed in the study by Ntusi et al., showing a 65% higher prevalence of LGE in the PWH (95% CI: 55.0%–76.0%,), followed by Luetkens et al. with a 55% difference (95% CI: 31.0%–78.0%). Both were statistically significant. On the other hand, the study by Wu et al. showed the smallest and non-significant difference, with a 2% prevalence difference (95% CI: −6.0%–11.0%), indicating no significant difference in LGE prevalence between the groups (Figure 2). The substantial heterogeneity (I^2^ = 94.5%) across studies underscores the variability in prevalence estimates, suggesting that further research using standardized methodologies is necessary to clarify these differences. Furthermore, we conducted sensitivity analysis by sequentially excluding each study from the overall meta-analysis of pooled prevalence differences in LGE (Supplementary file 1 Table 4). This analysis demonstrated that the pooled prevalence difference remained robust, with values ranging from 26.4% to 39.2%. The exclusion of individual studies resulted in only minor variations in the pooled prevalence difference, indicating that the overall effect was not overly influenced by any single study. For instance, when the study by Shuldiner et al. was excluded, the pooled prevalence difference increased to 38.1% (95% CI: 15.3%–61.0%), while the exclusion of Ntusi et al. led to a slightly lower prevalence difference of 26.4% (95% CI: 9.2%–43.6%). This sensitivity analysis further reinforced the assumption that PWH have a significantly higher prevalence of myocardial fibrosis, as indicated by LGE, compared to HIV-uninfected individuals.

### 3.4 Meta-analysis of mean difference in myocardial native T1 mapping between PWH and HIV-uninfected

**Figure 3:**
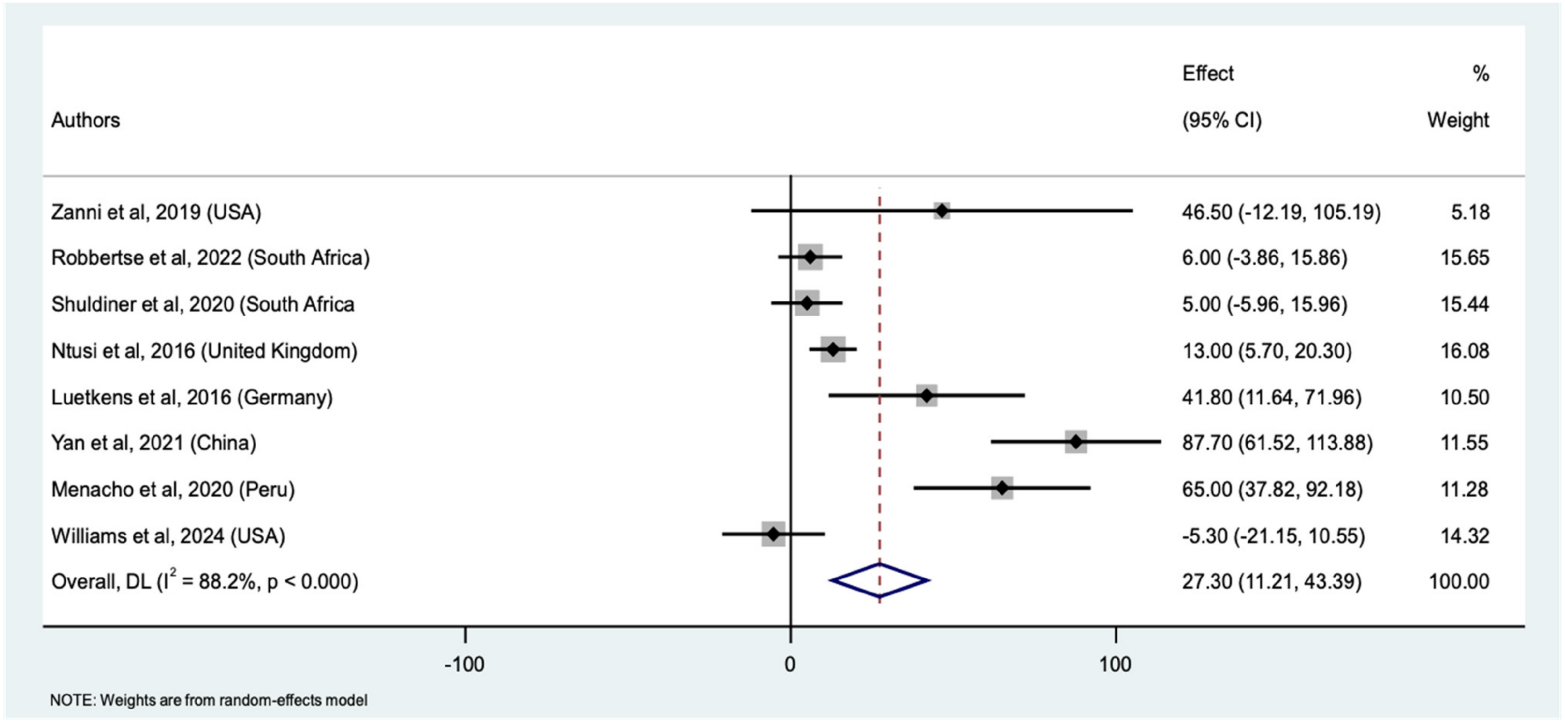
Forest plot of mean differences in native T1 mapping values between PWH and HIV-uninfected individuals across nine studies. The squares represent the effect sizes (mean difference) for each study, with horizontal lines indicating the 95% confidence intervals. The size of each square reflects the weight of the study in the meta-analysis. The diamond represents the overall pooled mean difference and its confidence interval, derived from a random-effects model.

Eight studies comparing native T1 mapping values between PWHand HIV-uninfected individuals were included in the meta-analysis. This yielded a total sample size of 840 participants (467 PWH and 373 HIV negative) (Supplementary file 1 Table 5). The pooled mean difference in native T1 mapping values between PWH and HIV-uninfected individuals was 27.30 ms (95% CI: 11.21, 43.39 ms, *p* < 0.001) (Figure 3). This significant difference indicates that PWH have higher native T1 mapping values compared to the HIV-uninfected individuals. Similarly, the weights assigned to individual studies were reflective of the number of PWH. The study by Ntusi et al. contributed the highest weight (16.1%) while Zanni et al. contributed the least weight (5.2%) to the pooled mean differences between the PWH and HIV-uninfected individuals. The individual study estimates varied considerably. For instance, the calculated mean difference for Zanni et al. was 46.50 ms (95% CI: −12.19, 105.19 ms), while Robbertse et al. and Shuldiner et al. had smaller differences of 6.00 ms (95% CI: −3.87, 15.87 ms) and 5.00 ms (95% CI: −5.96, 15.96 ms), respectively. The studies by Ntusi et al., Luetkens et al., Yan et al., and Menacho et al. showed significant mean differences of 13.00 ms (95% CI: 5.70, 20.30 ms), 41.80 ms (95% CI: 11.64, 71.96 ms), 87.70 ms (95% CI: 61.52, 113.89 ms), and 65.00 ms (95% CI: 37.82, 92.18 ms), respectively. With the highest mean difference observed by Yan et al. Conversely, Williams et al. had a negative mean difference of −5.30 ms (95% CI: −21.15, 10.55 ms), indicating even lower, but not statistically significant native T1 mapping values in PWH compared to the HIV-uninfected individuals. The forest plot (Figure 3) illustrates the individual study estimates and the overall pooled effect size for all the nine studies. The high heterogeneity among the studies (I^2^ = 88.2%%, *τ*^2^ = 405.3) indicates substantial variability in the mean differences, likely due to differences in study populations, methodologies, and settings. Despite this heterogeneity, the overall random-effects model confirms a significant pooled effect size. Sensitivity analysis, which involved systematically excluding each study, showed that the pooled mean difference ranged from 16.62 ms to 32.96 ms (Supplementary file 1 Table 6). These findings also supported increased myocardial fibrosis by native T1 mapping in PWH.

### 3.5 Meta-analysis of mean difference in ECVF in PWH and HIV-uninfected

**Figure 4:**
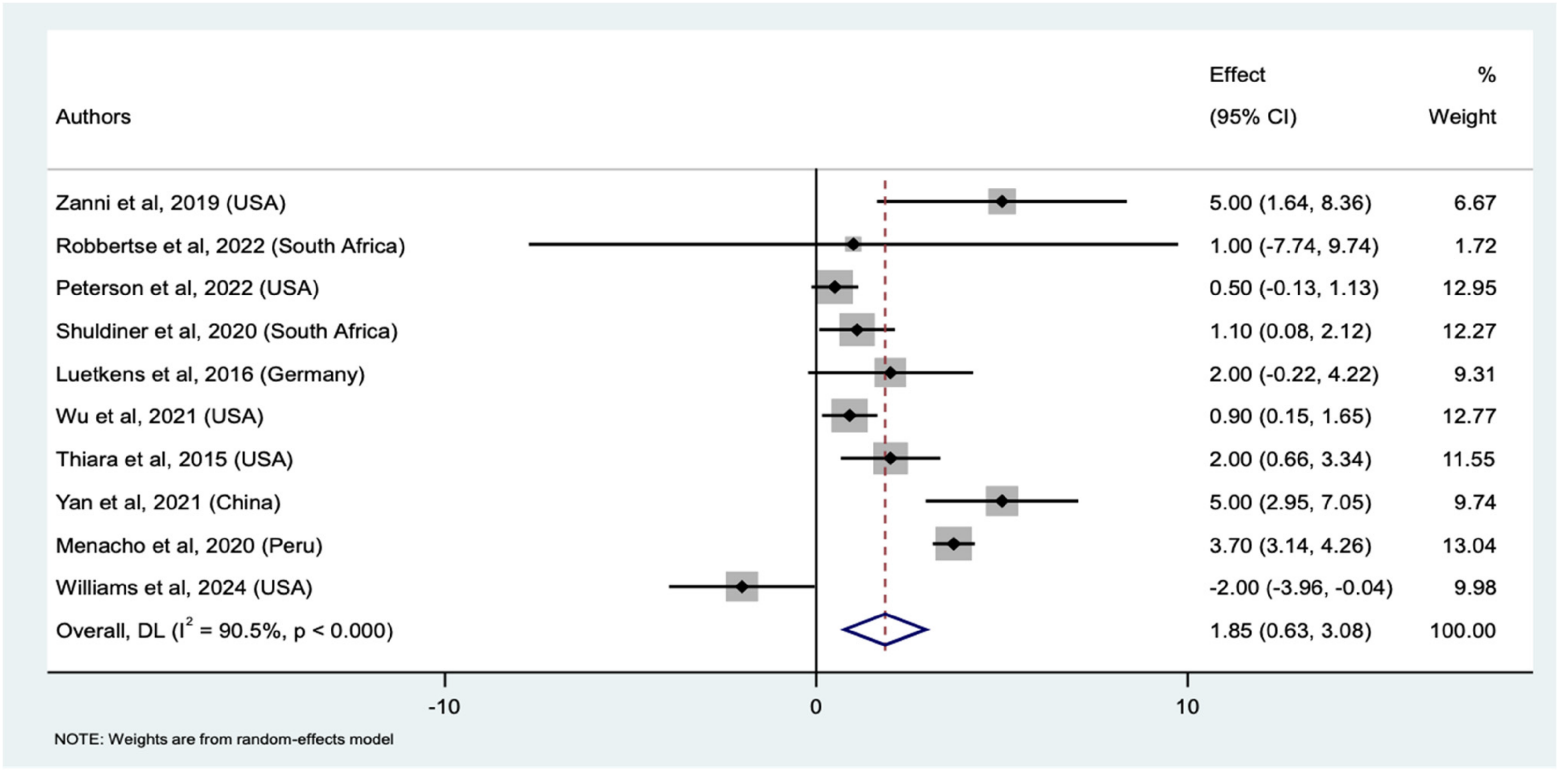
Forest plot of mean differences in ECVF between PWH and HIV-uninfected individuals across ten studies. The squares represent the effect sizes for each study, with horizontal lines indicating the 95% confidence intervals. The size of each square reflects the weight of the study in the meta-analysis. The diamond represents the overall pooled effect size which is the mean difference and its confidence interval, derived from a random-effects model.

We identified 10 studies that compared myocardial fibrosis between PWH and HIV-uninfected participants using ECVF (Supplementary file 1 Table 7), with a total sample size 1,603 (992 PWH and 611 HIV-uninfected). The pooled mean difference in ECVF between PWH and uninfected individuals was 1.85% (95% CI: 0.63%–3.08%, *p* < 0.001). This significant difference suggests increased ECVF in PWH compared to HIV-uninfected individuals. The weights assigned to each study were reflective of their respective proportion of PWH. The weights ranged from a low of 1.72% (Robbertse et al.) to a high of 13.04% (Menacho et al.), indicating varying degrees of influence among the studies analyzed. Individual study estimates also varied here. With Zanni et al., Yan et al., and Menacho et al. we found significant mean differences of 5.00% (95% CI: 1.64%–8.36%), 5.00% (95% CI: 2.95%–7.05%), and 3.70% (95% CI: 3.14%–4.26%), respectively. Conversely, Williams et al. indicated a significant negative mean difference of −2.00% (95% CI: −3.96% to −0.04%), indicating lower ECVF in PWH in that study. Other studies, such as those by Robbertse et al., Shuldiner et al., Peterson et al., and Luetkens et al., did not show statistically significant differences in ECVF values. The forest plot (Figure 4) illustrates the individual study estimates and the overall pooled mean difference. Again, we observed substantial heterogeneity (I^2^ = 90.5%, *τ*^2^ = 2.92), but the overall random-effects model confirms a significant pooled mean difference, underscoring the increased ECVF in PWH compared to uninfected controls. Similarly, the sensitivity analysis revealed that excluding individual studies from the meta-analysis consistently produced significant pooled mean differences, ranging from 1.47 to 2.66% (Supplementary file 1 Table 8), indicating minimal influence from any single study on the pooled results.

### 3.6 Meta-regression analysis of myocardial fibrosis severity using ECVF based on selected demographic and clinical factors among the PWH

We conducted meta-regression analyses to explore the influence of various demographic and clinical covariates on the severity of myocardial fibrosis, as indicated by ECVF, among PWH. The dependent variable was the severity of ECVF based on ECVF quantification. In the first meta-regression model, we included age, proportions of PWH (males and females), LVEDV, and LVEF based on the selection criteria under the methodology section. The results indicated that none of these covariates significantly predicted the severity of ECVF [Model F(5,1) = 0.43, *p* = 0.8116]. The Residual Maximum Likelihood (REML) estimate of τ^2^ was zero, indicating no residual heterogeneity, and the residual variation due to heterogeneity (I^2^_res_) was 0.00%. The individual coefficients for age (*p* = 0.574), proportions of males (*p* = 0.707), proportions of females (*p* = 0.864), LVEDV (*p* = 0.914), and LVEF (*p* = 0.612) were all non-significant. In the second meta-regression model, we incorporated total cholesterol, high-density lipoprotein (HDL) cholesterol, and low-density lipoprotein (LDL) cholesterol as covariates. Again, none of these covariates were found to significantly predict the severity of ECVF [Model F(3,3) = 0.67, *p* = 0.622], with τ^2^ at zero and I^2^_res_ at 0.00%. The coefficients for total cholesterol (*p* = 0.748), HDL cholesterol (*p* = 0.975), and LDL cholesterol (*p* = 0.830) were all non-significant. In the third meta-regression model, we examined the duration of ART and CD4 count as covariates. The results showed that neither ART duration nor CD4 count significantly predicted the severity of ECVF [Model F(2,2) = 0.90, *p* = 0.525]. The REML estimate of τ^2^ was zero, and I^2^_res_ was 0.00%. The coefficients for ART duration (*p* = 0.943) and CD4 count (*p* = 0.848) were both non-significant. These analyses suggest that the examined covariates did not significantly influence the severity of ECVF in PWH. The results are as shown in Table 2.

**Table 2:** Meta-regression analysis of myocardial fibrosis severity based on selected demographic, clinical, and HIV factors among the HIV infected.

Covariate	Coefficient	Standard error	*t*-value	*P*-value	95% confidence interval
Model 1					
Age	0.33	0.41	0.79	0.574	−4.94–5.59
Proportions of males	0.03	0.06	0.50	0.707	−0.79–0.85
Proportions of females	−0.02	0.09	−0.22	0.864	−1.10–1.06
LVEDV	0.01	0.10	0.14	0.914	−1.24–1.27
Ejection fraction	−0.52	0.75	−0.70	0.612	−10.05–9.01
Constant	44.28	29.99	1.48	0.379	−336.75–425.31
Model 2					
ART duration	0.11	1.41	0.08	0.943	−5.95–6.17
CD4 count	0.01	0.06	0.22	0.848	−0.26–0.28
Constant	19.73	23.45	0.84	0.489	−81.15–120.62
Model 3					
Total cholesterol	7.65	21.70	0.35	0.748	−61.41–76.71
HDL cholesterol	1.07	31.24	0.03	0.975	−98.34–100.48
LDL cholesterol	−6.58	28.10	−0.23	0.830	−96.00–82.83
Constant	9.35	19.62	0.48	0.666	−53.08–71.79

### 3.7 Publication biasness

**Figure 5:**
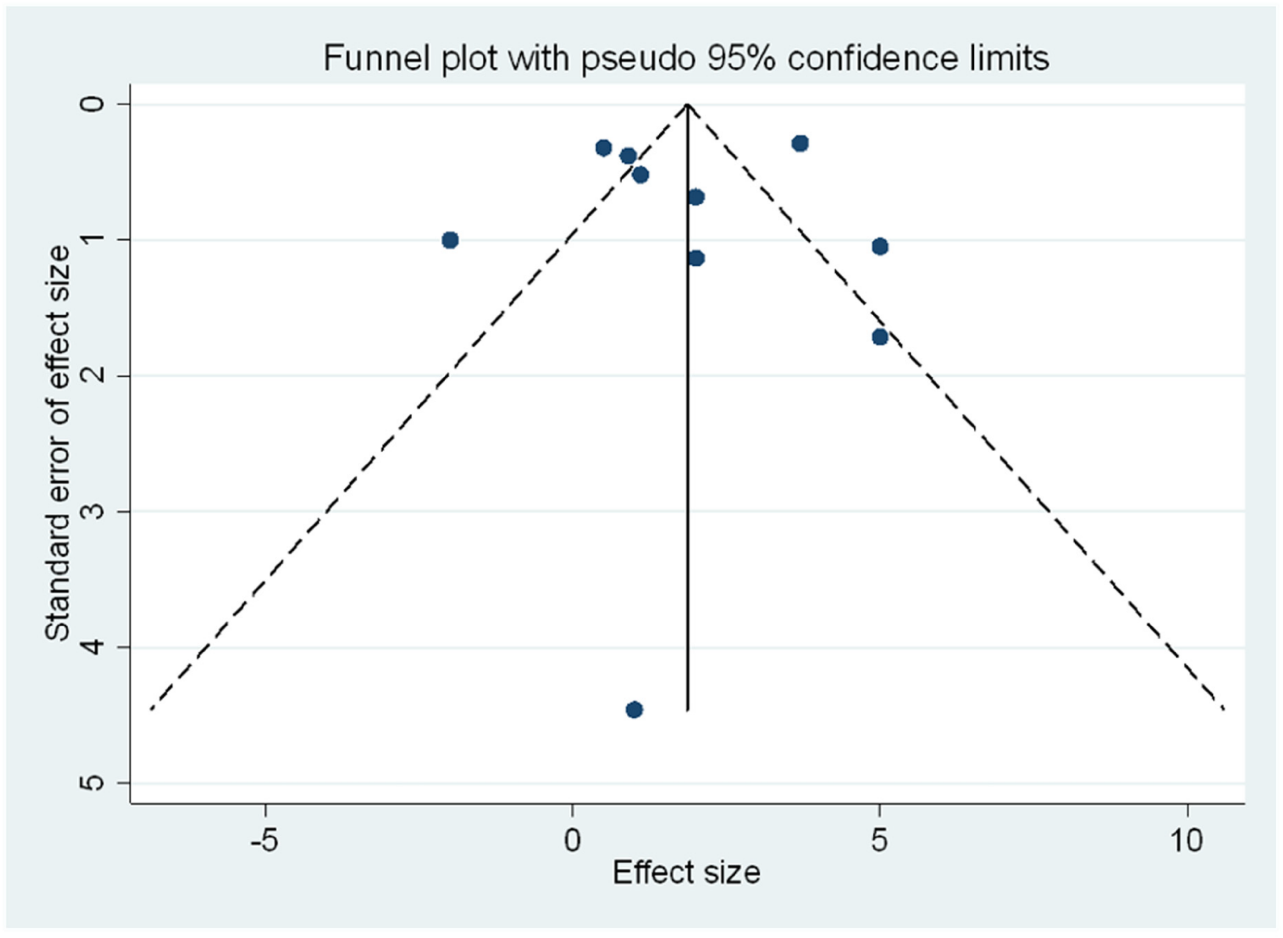
Funnel plot of the meta-analysis assessing publication bias. The plot shows the standard error of the effect size against the mean difference for ECVF for each study. The absence of asymmetry in the plot suggests no significant publication bias. The dashed lines represent the pseudo 95% confidence limits.

We only assessed publication bias in the studies looking at ECVF as they had reached a threshold of 10. The funnel plot (Figure 5) did not indicate significant asymmetry, suggesting the absence of publication bias. Egger's test further confirmed this, with a bias coefficient of −0.26 (95% CI: −4.85–4.33, *p* = 0.899), indicating no small-study effects. Additionally, we employed the trim-and-fill method which showed no need for trimming. The method did not identify any missing studies, as indicated by the unchanged pooled ECVF mean difference (Table 3). The fixed-effects model produced a pooled estimate of 1.87 (95% CI: 1.55–2.19, *z* = 11.42, *p* < 0.001), while the random-effects model yielded a pooled estimate of 1.85 (95% CI: 0.63–3.08, *z* = 2.962, *p* = 0.003). The consistency of these results before and after the application of the trim and fill method suggests that publication bias is unlikely to have significantly influenced the findings concerning ECVF, and further supported the data that the *p*-values presented for the pooled estimates showed the presence of a meaningful difference in ECVF percentage between the PWH and HIV-uninfected.

**Table 3:** Summary of the trim-and-fill method.

Method	Pooled E stimate	95% CI lower	95% CI upper	*z*-value	*p*-value	No. of studies
Fixed	1.87	1.55	2.19	11.422	<0.001	10
Random	1.85	0.63	3.08	2.962	0.003	10
Filled fixed	1.87	1.55	2.19	11.422	<0.001	10
Filled random	1.85	0.63	3.08	2.962	0.003	10

## 4 Discussion

Our systematic review and meta-analysis showed significant association between HIV infection and increased myocardial fibrosis, as evidenced by high prevalence of LGE, elevated native T1 mapping values, and ECVF in PWH compared to HIV-uninfected. The significantly high prevalence difference of LGE in the PWH compared to the HIV-uninfected group highlights the heightened burden of myocardial fibrosis in this population. This discrepancy suggests that HIV infection, even when managed with ART, might be associated with an increased risk of developing myocardial fibrosis, a condition that can have severe long-term cardiovascular implications. LGE is particularly adept at detecting replacement myocardial fibrosis, a type often associated with previous myocardial injury (14). In the context of HIV, the elevated prevalence of LGE-detected fibrosis suggests that subclinical myocardial damage might be more common than previously recognized, even in asymptomatic individuals.

This finding aligns with studies showing that PWH, despite effective ART and viral suppression, continue to experience chronic inflammation and immune activation, which may contribute to ongoing myocardial injury and fibrosis (2). The robust association between HIV and increased LGE prevalence emphasize the need for more vigilant cardiovascular monitoring in this population. Furthermore, the ability of LGE to detect subtle changes in myocardial tissue before they manifest clinically accentuates its value as a critical tool in the early identification of individuals at risk for adverse cardiovascular events, including heart failure and sudden cardiac death. The consistent findings across multiple studies in the meta-analysis reinforce the argument that myocardial fibrosis, as detected by LGE, is a significant and underappreciated complication of HIV infection, requiring integrating advanced imaging techniques like LGE-CMR into routine cardiovascular assessments for PWH.

The meta-analysis of native T1 mapping values also indicated the significant presence of myocardial fibrosis and inflammation in PWH compared to their HIV-uninfected counterparts. Native T1 times are particularly effective in detecting interstitial fibrosis, as they are independent of hematocrit levels, providing a more precise measure of myocardial tissue characteristics. However, it's noteworthy that the average native T1 times reported in many of the included studies were within normal limits (<1,050 ms for 1.5 T and <1,280 ms for 3 T machines) (Supplementary file 1 Table 5). This raises critical questions about whether the observed differences in native T1 times reflect clinically significant interstitial fibrosis in the context of HIV. Although these differences are statistically significant, their clinical implications warrant further exploration, particularly regarding the thresholds that define pathological fibrosis. Nonetheless, our findings resonate with those of the HIV post-mortem sudden cardiac death (HIV POST SCD) study by Tseng et al., which revealed a markedly higher burden of interstitial myocardial fibrosis among PWH who had arrhythmias compared to those without known HIV infection with arrythmias (3). The post-mortem evaluations in that study provide vital histologic confirmation of the diffuse myocardial changes suggested by imaging studies, such as native T1 mapping. Interestingly, while coronary artery disease has traditionally been considered the predominant cause of sudden cardiac death (SCD) in the general population, Tseng et al. found that only 23% of presumed SCDs in PWH individuals were due to coronary artery disease (3). Instead, interstitial myocardial fibrosis emerged as a significant pathological feature associated with sudden death from arrhythmias. This raises concern as to whether the CVD complications like arrhythmias may be triggered by diffuse myocardial fibrosis in PWH.

This finding is particularly relevant to our discussion, as it suggests that the elevated native T1 times observed in PWH may indeed reflect a clinically significant burden of interstitial fibrosis, potentially contributing to an increased arrhythmic risk and SCD. However, for these findings to be clinically actionable, there is a pressing need to establish specific thresholds using non-invasive methods like CMR to define clinically relevant interstitial fibrosis, as observed differences, while statistically significant, did not have wide margins. Our meta-analysis highlights this need and paves the way for future research to refine these thresholds and enhance the clinical utility of native T1 mapping in managing cardiovascular risks in PWH.

Similarly, the meta-analysis of studies comparing ECVF between PWH and HIV-uninfected individuals provides crucial insights into the extent of myocardial fibrosis associated with PWH. Elevated ECVF values, which indicate increased diffuse myocardial fibrosis, can lead to impaired cardiac function and heightened cardiovascular risks in this population. Our analysis particularly emphasized significant positive mean differences in ECVF reported by Zanni et al., Yan et al., and Menacho et al., suggesting that HIV infection is linked to diffuse myocardial fibrosis. For instance, Zanni et al. (21) found that asymptomatic, antiretroviral-treated women with HIV had increased myocardial fibrosis and reduced diastolic function compared to HIV negative women, with immune markers correlating with fibrosis severity. Yan et al. (20) similarly observed higher rates of myocardial fibrosis and inflammation among, particularly those with AIDS, highlighting the importance of early ART initiation. Menacho's study (25) also showed increased myocardial fibrosis and inflammation, along with higher prevalence of LGE, despite comparable baseline characteristics between PWH and control subjects.

However, there was notable variability in individual study estimates for ECVF, reflecting differences in study populations, methodologies, and clinical settings. For example, in our meta-analysis, Williams et al. (13) showed a significant negative mean difference of −2.00%, suggesting lower ECVF in PWH. A further review of this study, focusing on individuals with perinatally acquired HIV who had been on ART for decades, found no statistically significant differences in native T1 mapping and ECVF between PWH and control groups. The lack of observed fibrosis in this cohort is probably due to fewer conventional cardiovascular risk factors in the selected younger population, though the study's limitations - including small sample size, cross-sectional design, and use of historic controls- could also play a role. Additionally, it's important to consider hematocrit levels as a potential confounder in these findings. PWH often present with lower haematocrit due to lower haemoglobin levels (26), which could influence ECVF measurements. ECVF is calculated on CMR by measuring the difference between pre- and post-contrast T1 values in the myocardium and blood pool, adjusted for the patient's hematocrit, providing a quantitative assessment of the ECM volume relative to the total myocardial volume (16). Despite this, many studies did not compare hematocrit levels, limiting our ability to fully assess its impact. Therefore, the variability observed in ECVF estimates across studies emphasizes the need for future research with standardized methodologies and comprehensive reporting of confounders, such as hematocrit, to better understand the relationship between HIV infection and myocardial fibrosis.

Our meta-regression analysis attempted to identify potential demographic and clinical predictors of ECVF among PWH, including age, gender distribution, duration of ART, CD4 count, and various lipid measures. However, none of these variables showed a significant association with ECVF. Similarly, we did not find any associations between reported factors in the HIV-uninfected population. Conditions like hypertension, chronic kidney disease, diabetes mellitus, and hypercholesterolemia are known to drive diffuse myocardial fibrosis through mechanisms such as increased afterload, endothelial dysfunction, and chronic low-grade inflammation (27). These factors contribute to the overall myocardial remodeling observed in PWH, compounding the direct effects of the virus on the heart. It is crucial to consider these comorbidities when interpreting native T1 and ECVF findings, as they may confound the relationship between HIV and myocardial fibrosis, and more studies need to consider these traditional risk factors in interpretation of CMR diagnosed myocardial fibrosis. Unfortunately, we could not analyze these variables due to insufficient pooled data from the studies reporting these variables.

Overall, myocardial fibrosis represents a critical link between HIV infection and increased cardiovascular morbidity and mortality. Conditions such as sudden cardiac deaths among PWH have been attributed to myocardial fibrosis (3). The development of myocardial fibrosis among PWH has also been associated with diastolic dysfunction (21), potentially progressing to heart failure. Regular cardiovascular assessments, including imaging studies to detect early signs of myocardial fibrosis, should be integrated into routine care for PWH. Early detection of myocardial can facilitate timely interventions aimed at mitigating progression to overt cardiac dysfunction. However, further research is needed to elucidate the exact pathways through which HIV and its treatment contribute to myocardial fibrosis. Understanding these mechanisms is essential for developing targeted strategies to prevent and manage myocardial in this population. Potential areas of investigation include the role of specific inflammatory mediators, the impact of different ART regimens on myocardial tissue, and the identification of genetic or environmental factors that may predispose PWH to myocardial fibrosis.

### 4.1 Limitations

Our meta-analysis provided valuable insights but is subject to certain limitations. The observed high heterogeneity might reflect unique differences study designs, populations, and methodologies which we could not identify. Sensitivity analyses were attempted, including variations in study designs, participant demographics, and methodological approaches, but these efforts did not sufficiently correct the heterogeneity. The wide CIs observed in these studies further emphasizes the necessity for more standardized CMR protocols and comprehensive data collection in future research to reduce variability and enhance the reliability of findings. The lack of significant predictors in our meta-regression analyses also indicates that the pathophysiology of myocardial fibrosis in PWH might be multifactorial and not fully explained by the variables studied. Future studies should incorporate a broader range of variables such as inflammatory markers, traditional risk factors, and larger sample sizes to better understand these relationships.

## 5 Conclusion

There was a significant association between HIV infection and increased myocardial fibrosis, as evidenced by a high prevalence of LGE in PWH. Furthermore, there were consistently observed significant differences in native T1 mapping values and ECVF in the PWH compared to HIV-uninfected. However, more studies are needed to address knowledge gaps in CMR diagnosed myocardial fibrosis which will be crucial for developing targeted interventions to mitigate the cardiovascular burden in this population.

## Data Availability

The original contributions presented in the study are included in the article/Supplementary Material, further inquiries can be directed to the corresponding author.
