# Supplementary material for: The influence of HIV infection on myocardial fibrosis diagnosed by cardiac magnetic resonance imaging in adults: a systematic review and meta-analysis of observation studies
Source: Front Cardiovasc Med. 2025 Jan 29;12:1534533. doi: 10.3389/fcvm.2025.1534533 (PMC11814457; doi:10.3389/fcvm.2025.1534533)
Supplement: Supplementary file 1 [file Table1.docx]

**SUPPLEMENTAL MATERIAL**

**The influence of HIV infection on myocardial fibrosis diagnosed by cardiac magnetic resonance imaging in adults: a systematic review and meta-analysis of observation studies.**

**Katongo Hope Mutengo^1,8^, Bruno Bezerra Lima^2^, Wilbroad Mutale^3^, Aggrey Mweemba^4^, Lorrita Kabwe^5^, Clive Banda^6^, Callistus Kaayunga^7^, Mutale Mulenga^1^, Douglas Heimburger^7^, Sepiso Masenga^8^**†**, John Jeffrey Carr^9^** †**, Annet Kirabo^10^**†

^1^ Ministry of Health, Monze Mission Hospital, Department of Internal Medicine, Monze, Southern Province, Zambia;

^2^ Vanderbilt University Medical Center, Division of Cardiology, Nashville, TN, USA;

^3^ University of Zambia, School of Public Health, Lusaka, Zambia;

^4^ Ministry of Health, University Teaching Hospital Lusaka Adult Hospital, Nephrology Unit, Lusaka, Zambia;

^5^ Ministry of Health, National Heart Hospital, Department of Adult Cardiology, Lusaka, Zambia;

^6^ Ministry of Health, Southern Province Health Office, Choma, Southern Province, Zambia;

^7^ Vanderbilt University Medical Center, Vanderbilt Institute for Global Health, Nashville, TN, USA;

^8^ Mulungushi University School of Medicine and Health Sciences, HAND Research Group, Livingstone, Southern Province, Zambia;

^9^ Vanderbilt University Medical Center, Department of Radiology and Radiological Sciences, Nashville, TN, USA;

^10^ Vanderbilt University Medical Center, Department of Molecular Physiology and Biophysics, Nashville, TN, USA

**Equal Contributions: Sepiso Masenga^7^** †**, John Jeffrey Carr^8^** †**, Annet Kirabo^9^**† These authors contributed equally to the supervision of this work and share last authorship.

Corresponding Author: [hope.mutengo@moh.gov.zm](mailto:hope.mutengo@moh.gov.zm), [https://**orcid**.org/0000-0002-5039-9430](https://orcid.org/0000-0002-5039-9430)

**Supplemental Table 1 CMR scanners used in the assessment of outcomes with magnetic strength and protocols**

| **Author** | **CMR type** | **Native T1 mapping** | **Contrast agent for LGE** |
| --- | --- | --- | --- |
| Zanni et al | 3T Skyra, Siemens | Looker- Locker sequence | 0.15 mmol/kg of gadoterate meglumine, Dotarem |
| Robbertse et al | 1.5T Magnetom Avanto, Siemens Healthineers, Erlangen, Germany | modified-Look Locker inversion recovery (MOLLI) sequence | 0.2 mmol/kg of a macrocyclic gadolinium-based contrast agent |
| Shuldiner et al | 3T Skyra MR system, Siemens Healthcare | MOLLI sequence | 0.1 mmol/kg of gadoterate meglumine, Dotarem |
| Ntusi et al | 1.5T MR Avanto, Siemens Healthcare, Erlangen, Germany | MOLLI sequence | 0.1 mmol/kg of gadoterate meglumine, Dotarem |
| Luetkens et al | 3 T CMR system, Ingenia 3T; Philips Healthcare, Best, the Netherlands | MOLLI sequence | 0.2 mmol/kg of gadobutrol (Gadovist; Bayer Healthcare, Leverkusen, Germany) |
| Wu et al | 1.5T Siemens Avanto or Aera scanner, Erlangen, Germany | MOLLI sequence | 0.2 mmol/kg of gadobutrol (Gadavist^®^, Bayer, Montville, NJ, USA) |
| Thiara et al | 3.0T Verio; Siemens, Erlangen, Germany | MOLLI sequence | 0.15mmol/kg of Gadopentetate dimeglumine (Berlex; Bayer Healthcare, New Jersey) |
| Yan et al | 3.0T MAGNETOM Trio, Siemens Medical Systems, Erlangen, Germany | MOLLI sequence | 0.2 mmol/kg bodyweight Gadopentetate dimeglumine (Bayer Healthcare, New Jersey) |
| Menacho et al | 3T Magnetom, Prisma, Siemens, Siemens Medical Solutions, Erlangen, Germany. | MOLLI sequence | 0.1 mmol/kg of adoterate meglumine |
| Williams et al | 3T Siemens MAGNETOM Vida, Erlangen, Germany | Quantitatively assessed using the inline color map provided by the scanner manufacturer | 0.15 mmol/kg of Dotarem, Gurbet |
| Chew et al | 3.0T Siemens Trio, Siemens Medical Solutions, Erlangen, Germany | MOLLI sequence | 0.2 mmol/kg of Gadopentetate  dimeglumine |

**Supplemental Table 2 Quality assessment criteria**

| Author | Selection | Comparability | Exposure/Outcome | Overall |
| --- | --- | --- | --- | --- |
| Zanni et al | *** | ** | ** | 7 |
| Robbertse et al | **** | ** | *** | 9 |
| Peterson et al | **** | ** | *** | 9 |
| Shuldiner et al | *** | ** | ** | 7 |
| Ntusi et al | *** | ** | ** | 7 |
| Holloway et al | *** | ** | ** | 7 |
| Luetkens et al | **** | ** | ** | 8 |
| Wu et al | **** | ** | *** | 9 |
| Thiara et al | **** | ** | ** | 8 |
| Yan et al | *** | ** | ** | 7 |
| Menacho et al | *** | ** | ** | 7 |
| Williams et al | *** | ** | ** | 7 |
| Chew et al | *** | ** | * | 6 |

The meta-analysis study quality was assessed according to the Newcastle Ottawa Quality assessment scale for observational studies. ** 2 points. *** 3 points. **** 4 points.

**Supplemental Table 3 Summary of Studies Comparing LGE prevalence differences in PWH and HIV-uninfected**

| **Year** | **Study Type** | **Location** | **Author** | **Total Sample Size** | **PWH (n)** | **HIV-uninfected**  **(n)** | **LGE present**  **(PWH)**  **(n)** | **LGE present**  **(HIV-uninfected)**  **(n)** |
| --- | --- | --- | --- | --- | --- | --- | --- | --- |
| 2022 | Cohort | South Africa | Robbertse et al | 168 | 95 | 73 | 40 | 1 |
| 2020 | Cross-sectional | South Africa | Shuldiner et al | 229 | 134 | 95 | 93 | 65 |
| 2016 | Cross-sectional | United Kingdom | Ntusi et al | 195 | 103 | 92 | 84 | 15 |
| 2016 | Cohort | USA | Luetkens et al | 50 | 28 | 22 | 23 | 6 |
| 2021 | Cross-sectional | USA | Wu et al | 389 | 239* | 150* | 87 | 48 |
| 2021 | Cross-sectional | China | Yan et al | 77 | 47 | 30 | 10 | 0 |
| 2020 | Cross-sectional | Peru | Menacho et al | 47 | 26 | 21 | 20 | 0 |
| **Total** |  |  |  | **1,081** | **669** | **412** | **357** | **135** |

*Absolute numbers extrapolated from the percentages given in the original document

**Supplemental Table 4** **Sensitivity analysis of pooled prevalence difference in LGE percentages**

| **Study Excluded** | **Prevalence difference (%)** | **95% Confidence Interval** |
| --- | --- | --- |
| Robbertse | 30.7 | 7.5 to 53.9 |
| Shuldiner | 38.1 | 15.3 to 61.0 |
| Ntusi | 26.4 | 9.2 to 43.6 |
| Luetkens | 29.4 | 6.7 to 52.1 |
| Wu | 38.0 | 16.5 to 59.5 |
| Yan | 39.2 | 25.8 to 52.6 |
| Menacho | 31.7 | 7.2 to 56.2% |

This table illustrates the impact of excluding each study on the overall meta-analysis of pooled prevalence differences. Each exclusion slightly alters the pooled prevalence difference, yet the effect remains within a significant range, demonstrating the strength of the meta-analytic results despite the high heterogeneity among the studies, with values ranging between 26.4 to 39.2%

**Supplemental Table 5 Summary of studies comparing myocardial T1 mapping between PWH and HIV-uninfected**

| **Year** | **Study Type** | **Location** | **Author** | **Total Sample Size** | **PWH** | **HIV-uninfected** | **Native T1 mapping, ms (PWH)** | **Native T1 mapping, ms (HIV-uninfected)** |
| --- | --- | --- | --- | --- | --- | --- | --- | --- |
| 2019 | Cross-sectional | USA | Zanni et al | 34 | 20 | 14 | 1011.2 | 964.7 |
| 2022 | Cohort | South Africa | Robbertse et al | 168 | 95 | 73 | 1014 | 1008 |
| 2020 | Cross-sectional | South Africa | Shuldiner et al | 229 | 134 | 95 | 1247 | 1242 |
| 2016 | Cross-sectional | United Kingdom | Ntusi et al | 195 | 103 | 92 | 969 | 956 |
| 2016 | Cohort | USA | Luetkens et al | 50 | 28 | 22 | 1128.3 | 1086.5 |
| 2021 | Cross-sectional | China | Yan et al | 77 | 47 | 30 | 1337.2 | 1249.5 |
| 2020 | Cross-sectional | Peru | Menacho et al | 47 | 26 | 21 | 1295 | 1230 |
| 2024 | Cross-sectional | USA | Williams et al | 40 | 14 | 26 | 1230.1 | 1235.4 |
| **Total** |  |  |  | **840** | **467** | **373** |  |  |

Myocardial T1 mapping across diverse geographical locations including the USA, South Africa, the United Kingdom, China, and Peru. The studies are predominantly cross-sectional, with two cohort studies conducted in the USA and South Africa.

**Supplemental Table 6 Sensitivity analysis of pooled mean difference in native T1 mapping values**

| **Study Excluded** | **Pooled Mean Difference (ms)** | **95% Confidence Interval (ms)** |
| --- | --- | --- |
| Zanni | 26.27 | 9.70 to 42.84 |
| Robbertse | 32.42 | 12.21 to 52.64 |
| Shuldiner | 32.34 | 12.71 to 51.97 |
| Ntusi | 31.85 | 9.80 to 53.90 |
| Luetkens | 27.30 | 11.20 to 43.40 |
| Yan | 16.62 | 4.37 to 28.87 |
| Menacho | 21.65 | 6.19 to 37.12 |
| Williams | 32.96 | 15.17 to 50.76 |

This table displays the impact of excluding each of the mentioned study on the overall pooled mean difference in T1 mapping values between PWH and HIV-uninfected individuals. The results indicate that the pooled mean difference remains significant across all scenarios, with values ranging from 15.04 ms to 28.72 ms.

**Supplemental Table 7 Summary of studies comparing ECVF between HIV infected and HIV-uninfected**

| **Year** | **Study Type** | **Location** | **First Author** | **Total Sample Size** | **PWH** | **HIV-uninfected** | **ECVF % (PWH)** | **ECVF %**  **(HIV-uninfected)** |
| --- | --- | --- | --- | --- | --- | --- | --- | --- |
| 2019 | Cross-sectional | USA | Zanni | 34 | 20 | 14 | 34.0 | 29.0 |
| 2022 | Cohort | South Africa | Robbertse | 168 | 95 | 73 | 25.0 | 24.0 |
| 2022 | Cohort | USA | Peterson | 381 | 235 | 146 | 28.7 | 28.2 |
| 2020 | Cross-sectional | South Africa | Shuldiner | 229 | 134 | 95 | 30.4 | 29.3 |
| 2016 | Cohort | USA | Luetkens | 50 | 28 | 22 | 28.1 | 26.1 |
| 2021 | Cross-sectional | USA | Wu | 436 | 273 | 163 | 29.2 | 28.3 |
| 2015 | Cross-sectional | USA | Thiara | 125 | 95 | 30 | 28.0 | 26.0 |
| 2021 | Cross-sectional | China | Yan | 77 | 47 | 21 | 33.5 | 28.5 |
| 2020 | Cross-sectional | Peru | Menacho | 72 | 51 | 21 | 28.5 | 24.8 |
| 2024 | Cross-sectional | USA | Williams | 40 | 14 | 26 | 25.5 | 27.5 |
|  | Total |  |  | **1,603** | **992** | **611** |  |  |

The table details the research from various global locations, notably the USA, South Africa, China, and Peru, spanning from 2015 to 2024. It highlights the prevalence of cross-sectional studies in the dataset, with cohort studies also notably present, particularly in the USA and South Africa. The studies collectively involve 1,612 participants.

**Supplemental Table 8 Sensitivity analysis of pooled mean difference in effect sizes**

| **Study Excluded** | **Pooled Mean Difference (%)** | **95% Confidence Interval** |
| --- | --- | --- |
| Zanni | 1.47 | 0.48 to 2.46 |
| Robbertse | 1.63 | 0.37 to 2.89 |
| Peterson | 1.84 | 0.53 to 3.15 |
| Shuldiner | 1.84 | 0.48 to 3.20 |
| Luetkens | 1.87 | 0.63 to 3.11 |
| Wu | 1.97 | 0.58 to 3.35 |
| Thiara | 2.00 | 0.58 to 3.43 |
| Yan | 2.06 | 0.71 to 3.40 |
| Menacho | 2.26 | 1.04 to 3.48 |
| Williams | 2.66 | 0.95 to 4.36 |

This table illustrates the impact of excluding each study on the overall meta-analysis of pooled mean differences. Each exclusion slightly alters the pooled mean difference, yet the effect remains within a significant range, demonstrating the strength of the meta-analytic results despite the high heterogeneity among the studies. The sensitivity analysis reinforces the conclusion that the observed effects are not dependent on any single study, thus enhancing confidence in the overall findings.
